# Phytochemical Study and *In Vitro* Biological Activities of *Hibiscus panduriformis* Burm. f. (Malvaceae), *Alternanthera pungens Kunth* (Amaranthaceae), and *Wissadula rostrata* (Schumach.) Hook. f. (Malvaceae)

**DOI:** 10.1155/2023/8289750

**Published:** 2023-12-22

**Authors:** Boly Rainatou, Belem-Kabré Wendkouni Leila M. Esther, Kaboré Boukaré, Compaoré Souleymane, Koala Moumouni, Ouédraogo Noufou

**Affiliations:** ^1^Institute of Research in Health Sciences, Research & Development Laboratory/Phytomedicines and Medicines, 03 PO 7047, Ouagadougou 03, Burkina Faso; ^2^Laboratory of Organic Chemistry and Applied Physic (LCOPA), Doctoral School of Sciences and Techniques, University Joseph KI-ZERBO, 03 BP 7021, Ouagadougou 03, Burkina Faso

## Abstract

The present study investigated the phytochemical content of *Hibiscus panduriformis*, *Alternanthera pungens*, and *Wissadula rostrata* and assessed their radical scavenging and anti-inflammatory properties. n-Hexane, dichloromethane (DCM), ethyl acetate, and methanol extracts were prepared from the powdered plant parts. The phytochemical analysis was performed using qualitative high-performance thin-layer chromatography, and polyphenols were quantified using well-established methods. The anti-inflammatory effect was by lipoxygenase inhibition, while the antiradical impact was evaluated through DPPH and ABTS radicals. Steroids, triterpenoids, flavonoids, and tannins were identified in the three plants. The highest phenolic content (95.67 ± 2.19 mg gallic acid equivalent/g) was obtained in the methanolic extract of *W. rostrata*, while the lowest was measured in *H. panduriformis*. *H. panduriformis* was found to be highly rich in flavonoids (61.22 ± 0.09 mg rutin equivalent/g), condensed tannins (62.53 ± 0.03 mg catechin equivalent/g), and hydrolyzable tannins (125.1 ± 1.02 mg tannic acid equivalent/g). The methanolic extract of *H. panduriformis* displayed the greatest antilipoxygenase activity with an IC_50_ value of 8.78 ± 1.05 *μ*g/mL. It should be noted that although a moderate to low effect was observed, the extracts were more likely to scavenge DPPH (IC_50_ values ranged from 0.106 ± 0.010 to 1 mg/mL) than ABTS radicals. There was a strong to moderate correlation between the antilipoxygenase and DPPH radical scavenging effects of the methanolic extracts and total phenolic content (antilipoxygenase, *r* = 0.7175; DPPH, *r* = 0.9376). Furthermore, it is worth noting that this is the first report investigating the phytochemical analysis and *in vitro* biological properties of *Hibiscus panduriformis*. The results highlighted the richness of this plant in polyphenols and demonstrated its high and moderate effects on lipoxygenase and DPPH radicals, respectively. To this intent, further *in vivo* and *in vitro* studies on this plant, along with exhaustive phytochemical analysis, are needed.

## 1. Introduction

The inflammation process results from a response to noxious stimuli, including microorganisms, toxic compounds, or injured cells [1]. Inflammation is, therefore, a defense mechanism used by the organism to remove harmful stimuli and stimulate tissue repair [1, 2]. Acute inflammatory response usually lasts for a few days and is obtained when the inflammatory triggers are eliminated with less tissue damage. Inflammation becomes chronic more frequently after the failure to eradicate foreign bodies during acute inflammation, leading to various chronic inflammatory diseases such as diabetes, cardiovascular diseases, cancer, and autoimmune diseases [1, 3, 4]. Inflammation triggers multiple signaling pathways leading to the stimulation and release of several inflammatory mediators, including cytokines, proteases, prostaglandins, and leukotrienes [1].

Furthermore, other important inflammation markers involve free radicals or reactive oxygen species (ROS) [1]. The primary role of ROS production by polymorphonuclear cells at the site of inflammation is to kill pathogens [5]. However, chronic inflammation promotes excessive ROS production, leading to oxidative stress because of an imbalance between ROS production and elimination by the body's antioxidant system [5–7].

Steroidal and nonsteroidal anti-inflammatory drugs are usually synthetic compounds used to fight or reduce inflammation in inflammation-related diseases [8]. However, using such drugs is linked to several adverse effects, leading most people to use medicinal plants to cure inflammation-associated diseases [8].

In most developing and developed countries, there is an enormous surge in the use of herbal medicine for disease prevention and treatment. Herbal-based drugs or medicinal plants are believed to promote healthier living than synthetic drugs [9].

*Alternanthera pungens* (*A. pungens*) Kunth (Amaranthaceae), *Wissadula rostrata* (*W. rostrata*) (Schumach.) Hook. f. (Malvaceae), and *Hibiscus panduriformis* (*H. panduriformis*) Burm. f. (Malvaceae) are three plants used against several diseases in Burkina Faso. *Alternanthera pungens* (*Syn. Alternanthera repens* O. Ktze.) is a creeping perennial herbaceous plant with a 10-15 cm long stem with hair [10, 11]. Like most *Alternanthera* species, *A. pungens* is traditionally used to treat inflammation, pain, infectious diseases, and gastric disorders [10, 11]. *Wissadula rostrata* (*Syn. Wissadula amplissima* (L.) R.E.Fr. var. *rostrata* (Schumach. & Thonn.)) is a perennial shrub found near inhabited places. In traditional medicine, *W. rostrata* is commonly used against insect bites, diarrhea, inflammation, and allergy [11, 12]. In Burkina Faso traditional medicine, parts for therapeutic use include leafy stems and buds for *A. pungens*. In contrast, roots, leaves, and flowers administered externally or internally were used for *W. amplissima* [11]. Contrary to these two plants, few studies have been dedicated to *Hibiscus panduriformis* (Malvaceae). Although various authors have studied the genus *Hibiscus* and reported its use in traditional medicine, there are practically no reports regarding the traditional use of *H. panduriformis*. According to the “Useful Tropical Plants Database,” *Hibiscus panduriformis* is an upright and branched subwoody plant, annual to perennial, of short lifespan, reaching up to 3 m high with stems, branches, and leaves velvety-tomentose [13]. The plant is mainly used as a fiber and ornamental source, and its flowers are eaten in Congo [13]. In South India, the leaves of *Hibiscus panduriformis* are used to repair bone fractures [14].

The present study was undertaken to assess the phytochemical analysis, radical scavenging, and lipoxygenase inhibitory activities of the three medicinal plants.

## 2. Materials and Methods

### 2.1. Chemicals and Apparatus

n-Hexane, dichloromethane, ethyl acetate, methanol, sodium carbonate, and 2,2-azino-bis(3-ethylbenzothiazoline-6-sulfonic acid) (ABTS) diammonium salt were purchased from Merck (Darmstadt, Germany). Dimethyl sulfoxide (DMSO), gallic acid, tannic acid, rutin, catechin, aluminum chloride, 2,2-diphenyl-1-picrylhydrazyl (DPPH), Folin-Ciocalteu reagent, hydrochloric acid, zileuton, Tween 20, linoleic acid, and soybean lipoxygenase (type I-B) enzyme were from Sigma-Aldrich (St. Louis, USA). Trolox was obtained from Fluke (France), and aqueous water was distilled in our laboratory. All other chemicals were of analytical grade.

The spectrophotometric measurements were carried out using a Shimadzu UV-Vis Spectrophotometer and microplate spectrophotometer (BioTek Epoch, USA). High-performance thin-layer chromatography (HPTLC) silica gel F_254_ plates (20 cm × 10 cm) from Merck (Darmstadt, Germany) were used to identify the main phytoconstituents from the plant's extracts.

### 2.2. Collection and Extraction of the Plant's Materials

**Figure 1:**
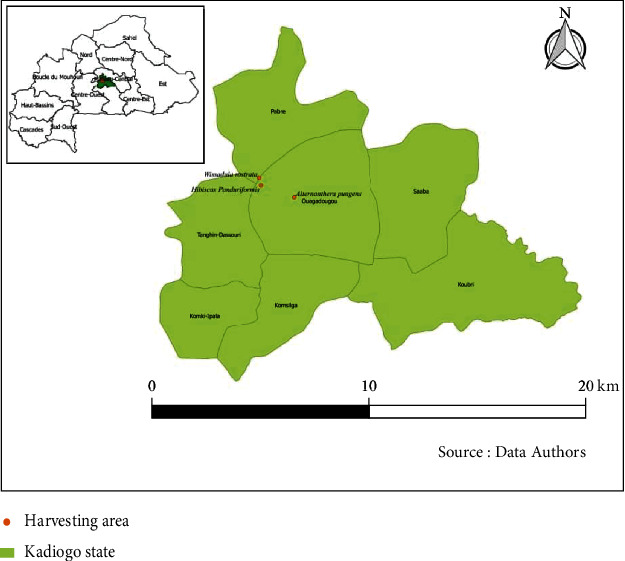
Location map showing the collection area of the plants.

The leafy stems of *Alternanthera pungens*, *Wissadula rostrata*, and *Hibiscus panduriformis* were collected from the periurban area around Ouagadougou, the capital city of Burkina Faso (Figure 1).

Each plant was taxonomically identified, and the voucher specimens were deposited in two different herbariums. *Wissadula rostrata* and *Alternanthera pungens* voucher specimens were deposited in the national herbarium of University Joseph KI-ZERBO under numbers 17259 and 17260, respectively. The *Hibiscus panduriformis* voucher specimen was deposited in the herbarium of the *Centre National de Semences Forestières* (CNSF, Burkina Faso) under reference number CNSF-1400.

The leafy stems of the plants were shade-dried at room temperature. Once dry, after two weeks, the plant's materials were powdered using a mechanic grinder. Extraction was done sequentially using solvents of increasing polarity, from n-hexane, dichloromethane (DCM), and ethyl acetate to methanol. The residue obtained in a previous extraction step was left to dry and extracted with successive solvents. Briefly, 100 g of each plant powder was first macerated with 500 mL of n-hexane under shaking for 24 hours. The extract was filtered, and the residue was resuspended in 500 mL of n-hexane for another 24 hours. Finally, this extraction is repeated once using the same solvent and following the same procedure. The n-hexane filtrates (to a volume approximately equal to 1.4 L) are mixed, and the residue is allowed to dry at room temperature. The different filtrates (n-hexane, DCM, ethyl acetate, and methanol (slightly acidified)) were concentrated using a rotary evaporator at 40°C. Each plant's hexane, DCM, ethyl acetate, and methanol extracts were conserved at 4°C for further analysis.

### 2.3. Qualitative High-Performance Thin-Layer Chromatography (HPTLC) Phytochemical Screening

The phytochemical analysis was performed on HPTLC silica gel 60 F_254_ plates to identify steroids, triterpenoids, flavonoids, and tannins [15]. 20 *μ*L (6 mg/mL) of samples was applied to the plate as 5 mm band length using the CAMAG Linomat 5 TLC sample applicator (CAMAG, Muttenz, Switzerland) equipped with a syringe. After application, the plates were developed vertically, ascending in a glass twin-trough chamber presaturated for 30 minutes with an adequate mobile phase. The chromatographic run length was 70 mm from the bottom edge of the plate. Following the development, the plates were dried using a hairdryer, and specific reagents were used to reveal the phytoconstituents. Flavonoids were detected with Neu's reagent; tannins were shown using a FeCl_3_ 2% reagent. To observe steroids and triterpenes, the plates were sprayed with the Liebermann-Burchard reagent. The plates were air-dried and heated at 110°C, except for those used to detect tannins, which were heated at 100°C [15].

### 2.4. Estimation of Total Phenolic Content (TPC)

The total polyphenol content was quantified spectrophotometrically using the Folin-Ciocalteu reagent (FCR) [16] with slight modifications. A calibration curve was realized with gallic acid concentrations ranging from 0.0001 to 0.5 mg/mL. The reaction mixture was prepared with 1 mL of plant extract solution (prepared from a stock solution of 1 mg/mL) and 1 mL of FCR (0.2 M). After 8 minutes at room temperature, 3 mL of 7.5% (*w*/*v*) sodium carbonate was added. The mixture was allowed to incubate for 30 min at room temperature, and the absorbance was read at 760 nm in a spectrophotometer (Shimadzu UV-Vis, Japan) against a blank sample (the extract was replaced by distilled water). Total phenolic content (TPC), expressed as milligram gallic acid equivalents per gram of dry extract weight (mg GAE/g), was calculated from the gallic acid calibration curve (*y* = 10.459*x* + 0.0335, *R*^2^ = 0.9993). The measurements were conducted in triplicate.

### 2.5. Estimation of Total Flavonoid Content (TFC)

The total flavonoid content (TFC) was determined using the aluminum chloride (AlCl_3_) colorimetric assay [17] with slight modifications. Briefly, 1 mL of methanolic extract of 1 mg/mL stock solution was mixed with 0.3 mL NaNO_2_ (5%). Then, 0.3 mL of 10% AlCl_3_ methanolic solution was added. Six minutes later, 2 mL NaOH (1 M) was added, and the total volume was made up to 5 mL with 2.4 mL of distilled water. The reaction mixture was left to stand at room temperature for 30 min, after which the absorbance was read at 510 nm with a Shimadzu UV-Vis (Japan) against a blank. Rutin was used as a standard for the calibration curve (*y* = 2.5608*x* + 0.0034, *R*^2^ = 0.9995), and total flavonoid content (TFC) was expressed as milligram rutin equivalents (mg RuE) per gram of dry extract weight of three independent experiments.

### 2.6. Determination of Condensed Tannin Content (CTC)

The vanillin/hydrochloric acid method was used to assess the condensed tannin content [18] in methanolic extracts of the three plants. Diluted solutions of plant extracts or catechin, the reference compound, were made from a 1 mg/mL stock solution. In cleaned glass tubes protected from light with aluminum foil, 0.5 mL of each sample (extract/catechin), 3 mL of a 4% methanolic vanillin solution, and 1.5 mL of concentrated hydrochloric acid were thoroughly mixed. Tubes were allowed to stand at room temperature for 20 min, and absorbances were recorded at 500 nm (Shimadzu UV-Vis, Japan) against a blank. The content of condensed tannin was estimated from the catechin calibration curve (*y* = 2.6248*x* + 0.0266, *R*^2^ = 0.9962) and expressed as milligram catechin equivalents (mg CE) per gram of dry extract weight.

### 2.7. Determination of the Hydrolyzable Tannin Content (HTC)

Hydrolyzable tannins are a group of natural polyphenols with a central core of glucose or another polyol being esterified with either gallic acid (the compounds are called gallotannins) or hexahydroxydiphenic acid (also called ellagitannins) [19]. The hydrolyzable tannin content of the methanolic extracts was determined using tannic acid as the reference compound for the calibration curve [20]. Diluted methanolic extracts and tannic acid were prepared from stock solutions (extract/standard) of 1 mg/mL. 1 mL of each solution was mixed with 5 mL of 2.5% KIO_3_. The mixture was vortexed and left to incubate for 4 minutes at room temperature. After that, the absorbance of the red-colored mixture was read at 550 nm (Shimadzu UV-Vis, Japan) against a distilled water blank. The hydrolyzable tannins were quantified from the tannic acid calibration curve (*y* = 0.4055*x* + 0.0213, *R*^2^ = 0.9973), and the results were expressed as milligram tannic acid equivalents per gram of dry weight (mg TAE/g).

### 2.8. Lipoxygenase (LOX) Inhibition Assay

The lipoxygenase inhibition assay was performed on a 96-well microplate with linoleic acid as substrate [21]. The test sample was dissolved in DMSO at 8 mg/mL, and diluted solutions were prepared. 3.75 *μ*L of each diluted solution was added to the reaction mixture containing 146.25 *μ*L of an aqueous solution of lipoxygenase (820.51 U/mL). The plate was allowed to incubate for three minutes at room temperature, and the reaction was initiated by adding 150 *μ*L of linoleic acid solution (1.25 mM). An enzyme blank without inhibitor was made of 153.75 *μ*L of borate buffer (0.2 M, pH = 9) and 146.25 *μ*L of an aqueous solution of lipoxygenase (820.51 U/mL). The sample blank (150 *μ*L of borate buffer + 3.75 *μ*L of the test sample and 146.25 *μ*L of enzyme) was also realized. Zileuton was used as a reference compound. Inhibitory activity was measured in triplicate using a microplate spectrophotometer (BioTek Epoch, USA) at 234 nm. The percent of lipoxygenase inhibition was obtained from the following equation:

(1) $$\begin{array}{c} \text{Inhibition }\left(\%\right)=\left[\frac{A_{\text{C}}-A_{\text{s}}}{A_{\text{C}}}\right]\times 100, \end{array}$$

where *A*_C_ is the absorbance of the enzyme—absorbance of enzyme blank—and *A*_S_ is the absorbance of the sample—absorbance of the sample blank.

### 2.9. Determination of the 2,2-DPPH Radical Scavenging Activity

The 2,2-DPPH^•^ radical scavenging activity was assessed using a Shimadzu UV-Vis Spectrophotometer [22]. Trolox and plant extracts were dissolved in methanol to yield a final 1 mg/mL concentration, from which diluted solutions were prepared. 1 mL of sample or reference compound methanolic solution was mixed with 4 mL of 0.004% methanolic DPPH work solution. The mixture was incubated in the dark at room temperature for 10 minutes. For the blank sample, extract/Trolox was replaced with 1 mL of methanol. Absorbances were recorded at 517 nm in a Shimadzu UV-Vis Spectrophotometer (Japan), and measurements were conducted in triplicate. The DPPH radical scavenging activity (%) was calculated as follows:

(2) $$\begin{array}{c} \text{DPPH scavenging activity }\left(\%\right)=\left[\frac{\left(A_{\text{B}}–A_{\text{S}}\right)}{A_{\text{B}}}\right]\times 100, \end{array}$$

where *A*_B_ and *A*_S_ represent, respectively, the absorbances of the blank and sample/Trolox.

The IC_50_ (mg/mL), sample concentration or standard compound that produces half-maximal inhibition, and antioxidant radical power (ARP) were determined. Usually, a lower IC_50_ value indicates a high radical scavenging effect [23].

(3) $$\begin{array}{c} \text{ARP}=\frac{1}{{\text{IC}}_{50}}. \end{array}$$

### 2.10. Determination of the 2,2-Azino-Bis(3-Ethylbenzothiazoline-6-Sulfonic Acid) Radical Scavenging Assay

The ABTS^•+^ was generated by reacting the ABTS (7 mM) salt with potassium persulfate (39.2 mM) according to the method of Re et al. [24]. The mixture was incubated in the dark for 12-16 hours at room temperature. Before use, the stock solution was diluted to obtain an absorbance equal to 0.7 at 734 nm. Diluted solutions were prepared from a stock solution (plant sample) of 4 mg/mL. 1 mL of the different solutions was added to 4 mL of the diluted ABTS^•+^ solution, and absorbances were read at 734 nm in a spectrophotometer (Shimadzu UV-Vis, Japan) after 10 minutes of incubation in the dark.

(4) $$\begin{array}{c} \text{ABTS scavenging activity }\left(\%\right)=\left[\frac{\left({\text{A}}_{\text{C}}–{\text{A}}_{\text{S}}\right)}{{\text{A}}_{\text{C}}}\right]\times 100, \end{array}$$

where *A*_C_ and *A*_S_ are, respectively, the absorbances of the control (ABTS radical solution without extract or Trolox) and sample/Trolox (ABTS radical + extract/Trolox).

As in the DPPH assay, IC_50_ (milligrams per milliliter) values estimated the ABTS radical scavenging effect.

### 2.11. Statistical Analysis

The analyses were conducted in triplicate, and the results were expressed as means ± standard deviation (SD). Pearson's coefficients were determined for the correlation study. As previously reported, the GraphPad Prism (version 8.0) was used to perform the statistical differences between the extracts and to compare the extracts to the control, using a two-way analysis of variance (ANOVA) followed by multiple comparison tests [25]. The difference was considered statistically significant for a *p* value < 0.05.

## 3. Results

### 3.1. HPTLC Phytochemical Screening

**Figure 2:**
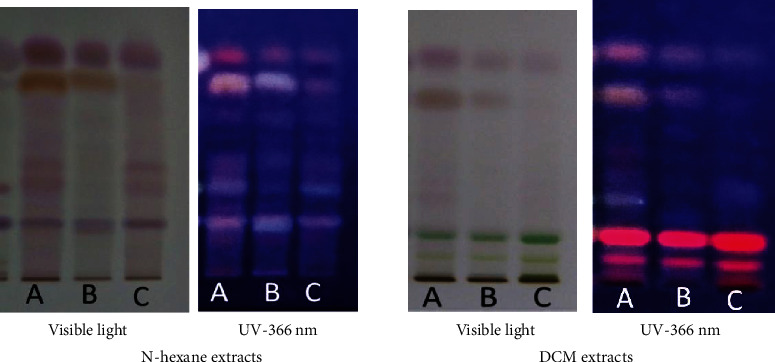
HPTLC profile for steroids and triterpenoids detected in the hexane and dichloromethane (DCM) extracts of (A) *Hibiscus panduriformis*, (B) *Alternanthera pungens*, and (C) *Wissadula rostrata*. The plates were developed into a chamber containing the mobile phase solvent system n-hexane/ethyl acetate (20 : 4, *v*/*v*).

**Figure 3:**
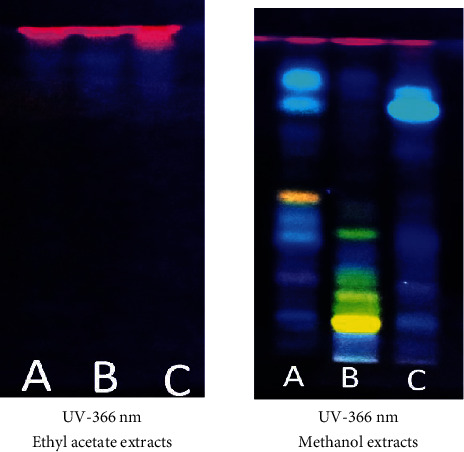
HPTLC chromatogram for flavonoids detected in the ethyl acetate and methanol extracts of (A) *Hibiscus panduriformis*, (B) *Alternanthera pungens*, and (C) *Wissadula rostrata*. The plates sprayed with the Neu reagent gave spots with orange, yellow, blue, and green colors, indicating the presence of flavonoids.

**Figure 4:**
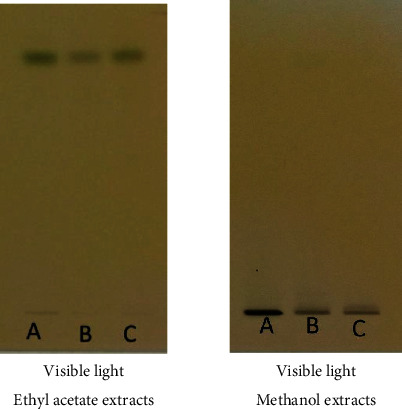
HPTLC chromatogram for tannins detected in the ethyl acetate and methanol extracts of (A) *Hibiscus panduriformis*, (B) *Alternanthera pungens*, and (C) *Wissadula rostrata*.

The main phytoconstituents of the three plants were analyzed using high-performance thin-layer chromatography (HPTLC). The technique identifies steroids, triterpenoids, flavonoids, and tannins (Figures 2–4).

The appearance of red-violet, blue, and green grass spots (Figure 2) revealed the presence of triterpenoids and steroids [26], respectively.

The HPTLC fingerprint (Figure 3) of flavonoids was obtained after development in the solvent system ethyl acetate/formic acid/water (80/10/10, *v*/*v*/*v*) for both the ethyl acetate and methanol extracts from the three plants. The ethyl acetate/formic acid/water (80/10/10, *v*/*v*/*v*) system appears more suitable for separating flavonoid compounds in the methanol extracts than the ethyl acetate extracts of the three plants.

Dark blue and greenish-black colors after derivatization at visible light highlight the presence of tannins (Figure 4) in the ethyl acetate and methanol extracts. The plates were developed in a chamber containing the following mobile phase solvent system: ethyl acetate-methanol-water-chloroform (18-2.4-2.1-6, *v*/*v*/*v*/*v*). Tannins were observed by spraying the plates with a 2% FeCl_3_ solution.

### 3.2. Total Phenolic, Flavonoid, Condensed Tannins, and Hydrolyzable Tannins

The total phenolic, flavonoids, condensed, and hydrolyzable tannins were determined in the methanol extracts of the three plants.  Table 1 presents the contents of polyphenolic compounds in the methanolic extracts of *H. panduriformis*, *A. pungens*, and *W. rostrata*.

Regarding the estimation of TPC, *Wissadula rostrata* showed the highest phenolic content (95.67 ± 2.19 mg GAE/g dry extract), followed by *Alternanthera pungens* (78.68 ± 1.60 mg GAE/g dry extract) and *Hibiscus panduriformis* (72.02 ± 0.71 mg GAE/g dry extract). Total flavonoid content can be ranked as follows: *H. panduriformis* (61.22 ± 0.09 mg RuE/g dry extract) > *W. rostrata* (21.77 ± 0.02 mg RuE/g dry extract) > *A. pungens* (8.83 ± 0.09 mg RuE/g dry extract). Condensed tannins were found to be rich in *H. panduriformis* (62.53 ± 0.03 mg CE/g dry extract), followed by *W. rostrata* and *A. pungens*. There was no statistically significant difference between *W. rostrata* and *A. pungens* concerning the condensed tannin content. *H. panduriformis* showed the maximum amount of hydrolyzable tannins (125.1 ± 1.02 mg TAE/g dry extract), followed by *A pungens* (37.82 ± 0.14 mg TAE/g dry extract) and *W. rostrata* with 15.43 ± 1.00 mg TAE/g dry extract.

### 3.3. Lipoxygenase (LOX) Inhibitory Activity

The results of lipoxygenase inhibitory activity by the organic solvent extracts are presented in Table 2.

The organic solvent extracts were highly statistically different (*p* < 0.0001). Comparatively to zileuton (*p* < 0.0001), the extracts from *H. panduriformis*, *A. pungens*, and *W. rostrata* showed high to weak effects with IC_50_ values ranging from 8.78 to 194.88 *μ*g/mL. The methanolic extract of *Hibiscus panduriformis*, with an IC_50_ value of 8.78 ± 1.05 *μ*g/mL, demonstrated good inhibitory activity compared to the other organic solvent extracts. With IC_50_ values greater than 200 *μ*g/mL, the hexane extract of *H. panduriformis* and methanolic extracts of both *A. pungens* and *W. rostrata* did not exhibit significant inhibitory activity. The inhibition percentages at this concentration (200 *μ*g/mL) were 47.90 ± 0.90, 49.27 ± 0.87, and 33.14 ± 1.54 *μ*g/mL, respectively, for the hexane extract of *H. panduriformis* and methanolic extracts of *A. pungens* and *W. rostrata*.

### 3.4. DPPH and ABTS Radical Scavenging Assay

The free radical scavenging ability of the organic solvent extracts was assessed using the DPPH and ABTS radical scavenging assays. The results are shown in Tables 3 and 4. The extracts were statistically highly (*p* < 0.0001) different regarding the two assays.

Overall, the results showed a weak radical scavenging effect of the different extracts on the two free radicals. The extracts were more active against DPPH than ABTS radicals. Trolox was at least thirty and five hundred times more potent than most extracts against DPPH and ABTS radicals, respectively (Table 3). The three plants' extracts, particularly the ethyl acetate and methanol extracts, were more effective on the DPPH radicals. In the two free radical scavenging tests and for each plant, there was a highly significant difference (*p* < 0.0001) between the different organic solvent extracts.

### 3.5. Correlation Studies

To find an eventual correlation between the three plants' *in vitro* biological properties and total polyphenol compounds, the Pearson correlation coefficient (*r*) and coefficient of determination (*R*) were obtained from GraphPad Prism. Some of these results are reported in Table 5.

A correlation coefficient equal to 0 means no linear relationship, while a negative or positive value indicates a negative or positive linear correlation. When the correlation coefficient value is between 0 and 0.3, 0.3 and 0.7, and 0.7 and 1, it means a weak, moderate, and strong correlation, respectively [25]. Based on these interpretations, it can be suggested that the DPPH radical scavenging effect is strongly positively correlated with TPC and strongly negatively correlated with the TFC, CTC, and HTC. Total phenolic content is moderately correlated (*r* = 0.6636) with the ABTS radical scavenging activity. There was a positive (0 < *r* ≤ 0.3) and a negative (−0.3 < *r* ≤ 0) weak correlation between ABTS radical scavenging effect and TFC (*r* = 0.2805), CTC (*r* = 0.0563), and HTC (*r* = −0.1489). The correlation study found a weak positive correlation (*r* = 0.3621) between the two radical scavenging methods.

Pearson's correlation coefficient between the antilipoxygenase activity and total phenolic content showed a weak (*r* = 0.2016) to moderate (*r* = 0.7175) correlation regarding the ethyl acetate and methanolic extracts. There were no significant correlations between TPC and the antilipoxygenase activity of the n-hexane (*r* = −0.4923) and DCM (*r* = −0.1348) extracts. Strong positive and moderate positive correlations were observed between the antilipoxygenase of the methanolic (*r* = 0.9149) and ethyl acetate (*r* = 0.5295) extracts and the DPPH radical scavenging effect. However, Pearson's correlation coefficient was weakly negative (*r* = −0.0449) between the ABTS assay and the antilipoxygenase of the methanolic extracts.

## 4. Discussion

Various medicinal plants have been used in folk medicine for their anti-inflammatory properties. Numerous *in vitro* and *in vivo* assays have been developed to assess the anti-inflammatory effect of plant samples [27]. Moreover, studies have reported the role of reactive oxygen species (ROS) in inflammation; therefore, scavenging ROS may increase the therapeutic strategy for inflammatory diseases [27]. The present study reports the lipoxygenase inhibitory activity, radical scavenging effect, and phytochemical analysis of *H. panduriformis*, *A. pungens*, and *W. rostrata*.

The phytochemical study has revealed the presence of steroids, triterpenes, and polyphenol compounds in the extracts of the three plants. Quantification of the polyphenolic compounds revealed that (i) phenolics were abundant in *W. rostrata* and (ii) flavonoids and condensed and hydrolyzable tannins were found to be rich in *H. panduriformis*. The results are consistent with previous reports [12, 28] regarding the total phenol content in *W. rostrata*. Polyphenol compounds present in *Alternanthera pungens* were mostly phenolic compounds, confirming other studies [10, 29]. Globally, the results indicate that the three plants have a high content of polyphenol compounds.

Lipoxygenases are oxidative enzymes that regulate inflammatory responses by generating proinflammatory or anti-inflammatory mediators known as leukotrienes and lipoxins [30, 31]. Based on their ability to incorporate oxygen atoms into arachidonic acid, LOXs are divided into four subtypes, including 5-LOX, 8-LOX, 12-LOX, and 15-LOX [32]. 5-LOX, 12-LOX, and 15-LOX have been implicated in several inflammation-related disorders such as atherosclerosis, asthma, and cancer [32]. Consequently, inhibiting LOX activity is crucial in the therapeutic strategy to alleviate these inflammatory disorders. The anti-inflammatory effect of the three plants was obtained through the *in vitro* inhibition of soybean 15-lipoxygenase. The results depicted in Table 1 indicate differences in their modes of LOX inhibition. The extracts may be classified as low, moderate, and highly active. n-Hexane extract of *H. panduriformis*, methanolic extracts of *A. pungens* and *W. rostrata*, DCM and ethyl acetate extracts of *A. pungens* showed weak to insignificant activity (IC_50_ > 100 *μ*g/mL) with respect to LOX inhibitory activity. n-Hexane extracts of *A. pungens* and *W. rostrata* and DCM and ethyl acetate extracts of *H. panduriformis* and *W. rostrata* exhibited moderate LOX inhibitory effect (10 *μ*g/mL ≤ IC_50_ < 100 *μ*g/mL). The methanolic extract of *H. panduriformis* can be classified as highly active against lipoxygenase (IC_50_ < 10 *μ*g/mL). Among the investigated extracts of each plant, the best LOX inhibition was obtained with the methanolic extract of *H. panduriformis*, n-Hexane extract of *A. pungens*, and DCM extract of *W. rostrata*. The results are consistent with other authors that have reported the anti-inflammatory activity, especially the antilipoxygenase activity of the Malvaceae family [28, 33, 34] and some species from the genus *Alternanthera* (Amaranthaceae) [10]. For instance, the ethanolic, hydroacetonic, and aqueous extracts of *W. amplissima* also developed a moderate (IC_50_ < 100 *μ*g/mL) inhibitory activity of lipoxygenase enzyme [28]. To the best of our knowledge, this is the first report about the antilipoxygenase activity of *Hibiscus panduriformis* and *Alternanthera pungens*. Medicinal plants contain numerous phytoconstituents that can be responsible for the good inhibitory effect of the three plant extracts. The phytochemical analysis has revealed the presence of steroids, triterpenes, and polyphenol compounds in the extracts of the three plants. These phytochemicals are known to elicit anti-inflammatory effects, primarily through the inhibition of lipoxygenase [26, 35, 36]. Pearson's correlation coefficient was used to find an eventual correlation between the anti-inflammatory activity and polyphenol compound contents. The results indicate that besides these compounds, other phytochemical compounds such as triterpenes and steroids probably contribute to the lipoxygenase inhibitory potential of the plants.

Recent evidence has stated the crucial role of reactive oxygen species (ROS) in the initiation, progression, and resolution of inflammation [37, 38]. Scavenging ROS contributes, therefore, to eliminate inflammation. The radical scavenging capacity of the extracts was evaluated using the radicals DPPH and ABTS. The results showed a low radical scavenging effect on the two radicals compared to Trolox. The methanolic extract of *H. panduriformis* gave higher DPPH scavenging activity than that of other organic solvent extracts. Although there are no reports on the pharmacological investigations of *H. panduriformis*, antioxidant activity has been reported on other *Hibiscus* species [39, 40]. Although weak, the DPPH scavenging effect of the ethyl acetate and methanolic extracts of *A. pungens* and *W. rostrata* was comparable to that of other previous reports [12, 28, 29]. The DPPH radical scavenging activity could have been underestimated. Various parameters, including DPPH concentration, plant extract composition, and incubation time, are involved in the DPPH radical scavenging activity [41]. In that study, DPPH was incubated with the extract for 10 minutes, while the literature reports an incubation time between 15 and 60 minutes. Only the methanolic extracts of the three plants exhibited a scavenging effect on the ABTS radical. However, this result was at least four hundred times less potent than Trolox.

It is well known that polyphenols are closely associated with antioxidant and anti-inflammatory activities of different medicinal plant parts [42, 43]. The weak positive correlation (*r* = 0.3621) between the DPPH and ABTS assays indicates differences in antiradical capabilities. The different extracts were likely more effective against DPPH radicals than ABTS ones (Tables 2 and 3), and the correlation studies revealed that the total phenolics contribute highly and moderately to the antiradical capacity of the three plants. However, based on earlier studies [43], this result does not mean that other polyphenol compounds, such as flavonoids and tannins, are not involved in the total free radical scavenging effect. Indeed, the antioxidant activity depends mainly on the structure and concentration of phenolic compounds, and polyphenol compounds can act (i) synergistically, (ii) additively, or (iii) antagonistically to eliminate free radicals [43]. Based on the correlation study between the anti-inflammatory effect and DPPH radical scavenging activity, it can be noticed that scavenging free radicals is involved in the anti-inflammatory property of the different extracts. Taken together, the results suggest that besides other phytochemicals, total phenolics are high contributors to the three plants' radical scavenging and antilipoxygenase properties.

## 5. Conclusions

The present study stated the phytochemical search, antilipoxygenase, and radical scavenging activities of *Hibiscus panduriformis*, *Alternanthera pungens*, and *Wissadula rostrata*. Findings revealed that total phenolics are mainly responsible for these activities. To the best of our knowledge, this is the first report on the antilipoxygenase and radical scavenging capacities of *H. panduriformis*. Further studies are needed to identify the polyphenols present in the extracts, mostly in *H. panduriformis*.

## Figures and Tables

**Table 1:** Polyphenol compound contents of the methanol extracts of the three plants.

Polyphenols	*Hibiscus panduriformis*	*Alternanthera pungens*	*Wissadula rostrata*
Phenolics^1^	72.02 ± 0.71^a^	78.68 ± 1.60^b^	95.67 ± 2.19^c^
Flavonoids^2^	61.22 ± 0.09^a^	8.83 ± 0.09^b^	21.77 ± 0.02^c^
Condensed tannins^3^	62.53 ± 0.03^a^	1.19 ± 0.09^b^	1.99 ± 0.01^b^
Hydrolyzable tannins^4^	125.1 ± 1.02^a^	37.82 ± 0.14^b^	15.43 ± 1.00^c^

Values presented are the average of three experiments ± standard deviation. ^1, 2, 3, 4^Expressed in terms of GAE, RuE, CE, and TAE (milligram of gallic acid, rutin, catechin, and tannic acid equivalents per gram of dry extract, respectively). In each row, values bearing different letters ^(a, b, and c)^ are considered highly statistically significant at *p* < 0.0001 (two-way ANOVA followed by Tukey's test).

**Table 2:** IC_50_ (*μ*g/mL) values of lipoxygenase (LOX) activity inhibition from the organic solvent extracts of the three plants.

IC_50_ (*μ*g/mL)				
Samples	*Hibiscus panduriformis*	*Alternanthera pungens*	*Wissadula rostrata*	*Zileuton*
Reference				3.11 ± 0.37
n-Hexane	> 200^a^	30.94 ± 0.32^d^	79.93 ± 0.76^h^	
Dichloromethane	35.10 ± 1.05^b^	194.88 ± 0.98^e^	54.55 ± 0.84^i^	
Ethyl acetate	36.18 ± 2.29^b^	149.84 ± 5.66^f^	85.75 ± 7.87^j^	
Methanol	8.78 ± 1.05^c^	> 200^g^	> 200^k^	

Values are presented as mean ± SD; each extract was tested in triplicate. For each plant, extracts were compared regarding the organic solvent (two-way ANOVA followed by Tukey's test) and zileuton (two-way ANOVA followed by Dunnett's test). Within each column, values bearing different letters ^(a, b, c, d, e, f, g, h, i, j, and k)^ are considered highly statistically significant at *p* < 0.0001. All organic extracts were statistically significantly (*p* < 0.0001) different from zileuton.

**Table 3:** DPPH and ABTS radical scavenging effect of different organic extracts of *H. panduriformis*, *A. pungens*, and *W. rostrata*.

Sample	DPPH IC_50_ (mg/mL)	ABTS IC_50_ (mg/mL)
*H. pan*-Hex	> 1^a^	> 4^a^
*H. pan*-DCM	> 1^a^	> 4^a^
*H. pan*-EtOAc	> 1^a^	> 4^a^
*H. pan*-MeOH	0.106 ± 0.010^b^	3.520 ± 0.006^b^
*A. pun*-Hex	> 1^a^	> 4^a^
*A. pun*-DCM	> 1^a^	> 4^a^
*A. pun*-EtOAc	0.926 ± 0.001^c^	> 4^a^
*A. pun*-MeOH	0.381 ± 0.027^d^	3.285 ± 0.019^c^
*W. ros*-Hex	> 1^a^	> 4^a^
*W. ros*-DCM	> 1^a^	> 4^a^
*W. ros*-EtOAc	0.485 ± 0.000^e^	> 4^a^
*W. ros*-MeOH	0.569 ± 0.014^f^	3.721 ± 0.286^d^
Trolox	0.012 ± 0.000^g^	0.007 ± 0.000^e^

Values are presented as mean ± SD, and each extract was tested in triplicate. For each test (DPPH and ABTS) and each plant, extracts were compared regarding the organic solvent (two-way ANOVA followed by Tukey's test) and Trolox (two-way ANOVA followed by Dunnett's test). For the DPPH test, values bearing different letters are considered highly statistically significant at *p* < 0.0001^(a vs. b, a vs. c, a vs. d, a vs. e, a vs. f, a vs. g, b vs. c, b vs. d, b vs. e, b vs. f, b vs. g, c vs. d, c vs. e, c vs. f, c vs. g, d vs. e, d vs. f, d vs. g, e vs. f, e vs. g, and f vs. g)^. For the ABTS test, values bearing different letters are considered highly statistically significant at *p* < 0.0001^(a vs. b, a vs. c, a vs. d, a vs. e, b vs. e, c vs. d, c vs. e, and d vs. e)^, at *p* = 0.0053^(b vs. c)^, and at *p* = 0.0165^(b vs. d)^. *H. pan: Hibiscus panduriformis*; *A. pun: Alternanthera pungens*; *W. ros: Wissadula rostrata*; Hex: hexane extract; DCM: dichloromethane extract; EtOAc: ethyl acetate extract; MeOH: methanol extract.

**Table 4:** Antiradical power (ARP) values of the DPPH and ABTS radical scavenging tests of different organic extracts of *H. panduriformis*, *A. pungens*, and *W. rostrata*.

Sample	DPPH ARP (1/IC_50_)	ABTS ARP (1/IC_50_)
*H. pan*-Hex	----	----
*H. pan*-DCM	----	----
*H. pan*-EtOAc	----	----
*H. pan*-MeOH	9.433	0.284
*A. pun*-Hex	----	----
*A. pun*-DCM	----	----
*A. pun*-EtOAc	1.080	----
*A. pun*-MeOH	2.625	0.304
*W. ros*-Hex	----	----
*W. ros*-DCM	----	----
*W. ros*-EtOAc	2.062	----
*W. ros*-MeOH	1.757	0.269
Trolox	83.333	142.857

ARP: antiradical power; *H. pan: Hibiscus panduriformis*; *A. pun: Alternanthera pungens*; *W. ros: Wissadula rostrata*; Hex: hexane extract; DCM: dichloromethane extract; EtOAc: ethyl acetate extract; MeOH: methanol extract. ----: not calculated.

**Table 5:** Correlations of IC_50_ values of DPPH and ABTS radical scavenging activity of the methanolic extracts of the three plants with total polyphenolic compounds.

Radical scavenging activity	Correlation *r*/*R*^2^				
	DPPH	TPC	TFC	CTC	HTC
DPPH	—	0.9376/0.8791	-0.7931/0.6290	-0.9103/0.8286	-0.9757/0.9519
ABTS	0.3621/0.1311	0.6636/0.4404	0.2805/0.0787	0.0563/0.0032	-0.1489/0.0222

*r*: correlation coefficient; *R*^2^: coefficient of determination. There was no statistically significant (*p* > 0.05) difference between the correlations. TPC: total phenolic content; TFC: total flavonoid content; CTC: condensed tannin content; HTC: hydrolyzable tannin content.

## Data Availability

All the data used are included in this manuscript.
